# The immune impact of mimic endoscopic retrograde appendicitis therapy and appendectomy on rabbits of acute appendicitis

**DOI:** 10.18632/oncotarget.16236

**Published:** 2017-03-15

**Authors:** Suqin Liu, Fenghua Pei, Xinhong Wang, Deliang Li, Lixia Zhao, Yanyan Song, Zhendong Chen, Bingrong Liu

**Affiliations:** ^1^ Department of Gastroenterology and Hepatology, The Second Affiliated Hospital of Harbin Medical University, Harbin, China; ^2^ Translational Medicine Research and Cooperation Center of Northern China, Heilongjiang Academy of Medical Sciences, Heilongjiang, China

**Keywords:** mimic endoscopic retrograde appendicitis therapy, appendectomy, intestinal immune function, TLR4/MYD88/NF-κB signaling pathway

## Abstract

This study was conducted to evaluate the immune impact of mimic endoscopic retrograde appendicitis therapy and appendectomy on rabbits of acute suppurative appendicitis and to determine whether TLR4/MYD88/NF-κB signaling pathway was activated in this process. 48 rabbits were assigned into 4 groups: group I, the mimic endoscopic retrograde appendicitis therapy group; group II, the appendectomy group; group III, the model group; and group IV, the blank group. White blood cells decreased, while levels of C-reactive protein, tumor necrosis factor-α, interleukin-6, interleukin-4, and interleukin-10 increased on the 2^nd^ day in group I and II. IgA in feces decreased at 2 weeks, while fecal microbiota changed at 2 and 4 weeks after appendectomy. CD8^+^ cells in appendix of group I increased within 8 weeks. Upregulated expression of TLR4, MYD88, and nuclear NF-κB were detected on the 2^nd^ day in group I and II. Mimic endoscopic retrograde appendicitis therapy and appendectomy are effective ways for acute suppurative appendicitis. Mimic endoscopic retrograde appendicitis therapy was more preferable due to its advantage in maintaining intestinal immune function. TLR4/MYD88/NF-κB signaling pathway was activated in acute phase of appendicitis.

## INTRODUCTION

Acute appendicitis is one of the most frequent underlying conditions in patients presenting with acute abdominal pain at the emergency department [1]. Previous studies showed that the annual rate of appendicitis was 10.776/10,000 in Taiwan from 2000 to 2011 and the overall incidence of acute appendicitis increased from 7.62 to 9.38 per 10,000 per year between 1993 and 2008 in the United States [2, 3]. Acute appendicitis might occur at any age. Acute appendicitis is classified into two categories: acute uncomplicated appendicitis and acute complicated appendicitis [4, 5]. The former consists of acute simple appendicitis and acute suppurative appendicitis; the latter kind includes gangrenous or perforated appendicitis and appendiceal abscess [4, 5].

The main treatments for acute appendicitis are appendectomy and antibiotics [6, 7]. In 1883, the first appendectomy was performed by Grooves in Canada. Three year later, surgeon Fitz clearly put forward that pericecal inflammation was caused by appendicitis, which made appendectomy advocated. Since then appendectomy has been widely adopted as the predominant treatment of acute appendicitis. However, this classic therapeutic treatment is somehow inducing postoperative complications, including wound infection, intra-abdominal infection, bleeding and intestinal obstruction [8-11]. In addition, negative appendectomy rate ranged from 5.4% to 17.6% [12-16]. Another side effect is the surgical scar, which may give rise to incisional hernia. Statistics suggested that antibiotic therapy was effective to 58.3%-73.4% of patients with acute uncomplicated appendicitis [17-20]. However, the recurrence rate of this treatment in 1 year was up to 15%-37% [17-20].

With the development of immunology and microbiology, people gradually realized that appendix plays a crucial role in intestine mucosal immunity and in maintaining the balance of intestinal flora [21-23]. To reduce the side effect and achieve minimally invasive treatment, in 2009, we established a new therapeutic method-endoscopic retrograde appendicitis therapy (ERAT) for acute uncomplicated appendicitis [24]. There are 5 steps in ERAT: (1) endoscopic appendiceal intubation; (2) appendiceal decompression; (3) retrograde appendicography; (4) stent drainage; and (5) cleansing the appendiceal lumen [24]. Our previous study demonstrated that effective rate of ERAT was up to 97%, with few complications [25]. To further analyze the impact of ERAT on immunity of intestine, we conducted the animal experiments to compare the immune function after treating acute suppurative appendicitis with mimic ERAT (Because colonoscopy can not be performed successfully on rabbit, the stent was placed in appendix surgically. The method was named mimic ERAT, mERAT for short.) or appendectomy and to determine whether TLR4/MYD88/NF-κB signaling pathway was activated in this process.

## RESULTS

**Figure 1:**
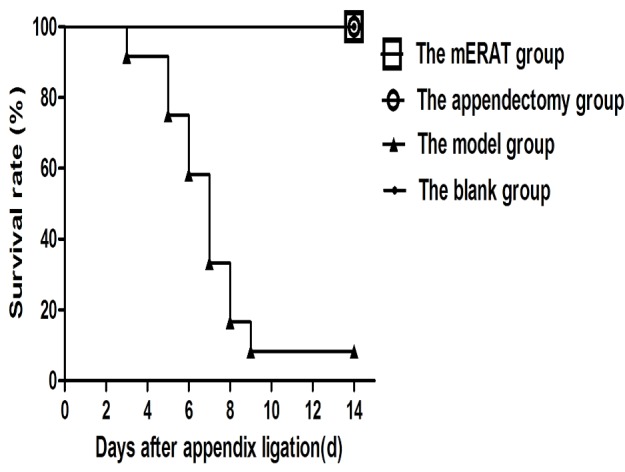
Survive curve

Rabbits of group I and II were mostly given water 1 day and food 2-3 days after treatment. Simultaneously, rabbits in group III continued with symptoms and became more and more serious. Finally, 11 rabbits died within 2 weeks while only one survived in group III (Figure 1).

**Figure 2:**
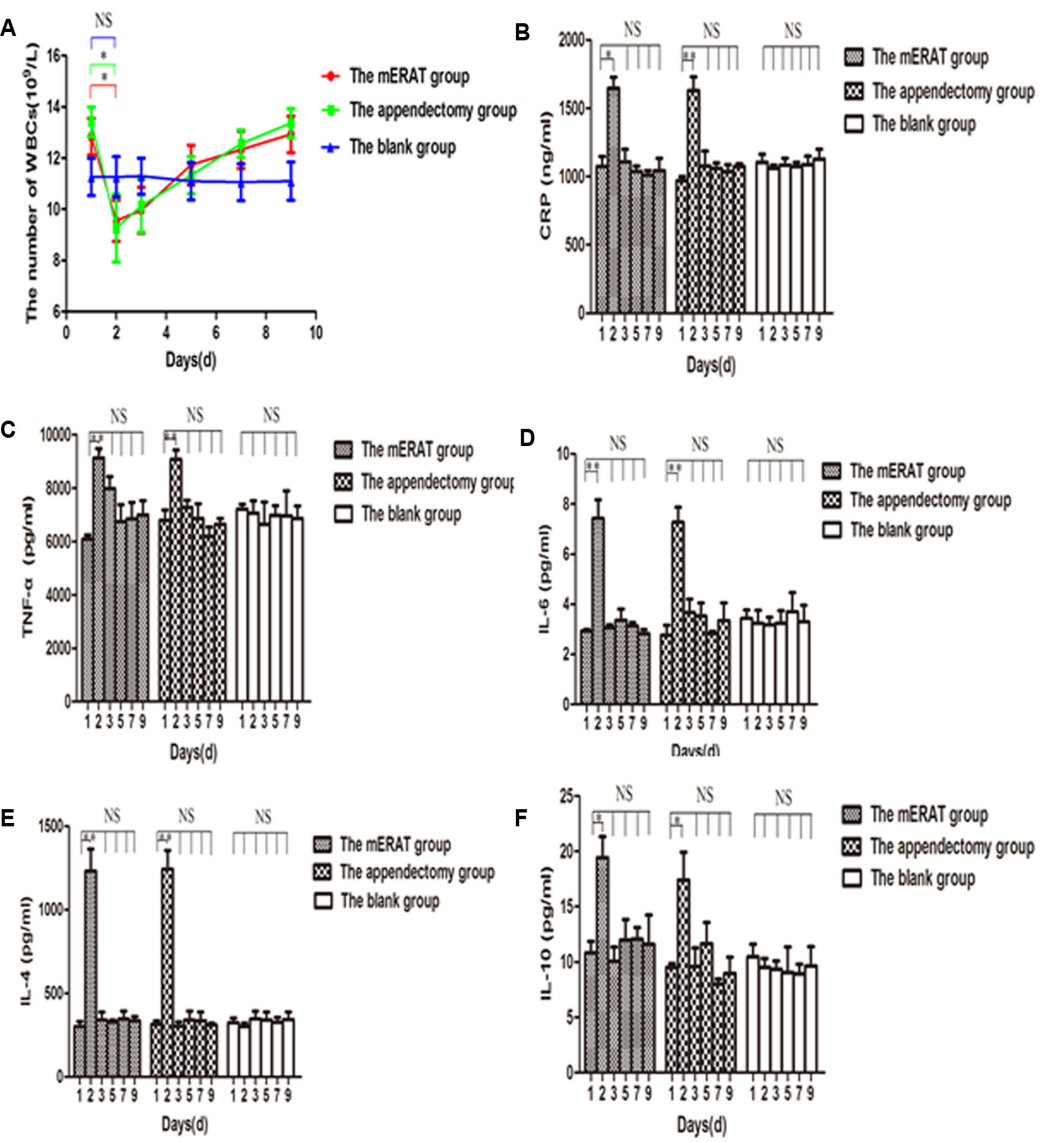
The trend of WBCs and inflammatory cytokines in rabbits of the mERAT group, the appendectomy group and the blank group on days 1, 2, 3, 5, 7, and 9 **A.** The number of WBCs. **B.** Levels of CRP were determined by Elisa. **C.** Levels of TNF-α. **D.** Levels of IL-6. **E.** Levels of IL-4. **F.** Levels of IL-10. (A-F) *P < 0.05, **P < 0.01, NS = no significance.

We have compared white blood cells (WBCs) count and several major inflammatory cytokines before and after treatment at the pointed time. The white blood cells (WBCs) decreased on the 2^nd^ day (after successful modeling), and returned to the original level on the 3^rd^ day in rabbits of group I and II (Figure 2A). Compared to the 1^st^ day, levels of C-reactive protein (CRP), tumor necrosis factor-α (TNF-α), interleukin-6 (IL-6), interleukin-4 (IL-4), and interleukin-10 (IL-10) went up on the 2^nd^ day (*P* < 0.05), and went back to the original level on the 3^rd^ day in group I and II (Figure 2B-2F). The trend of WBCs and inflammatory cytokines showed no significant difference between group I and II (*P >* 0.05).

**Figure 3:**
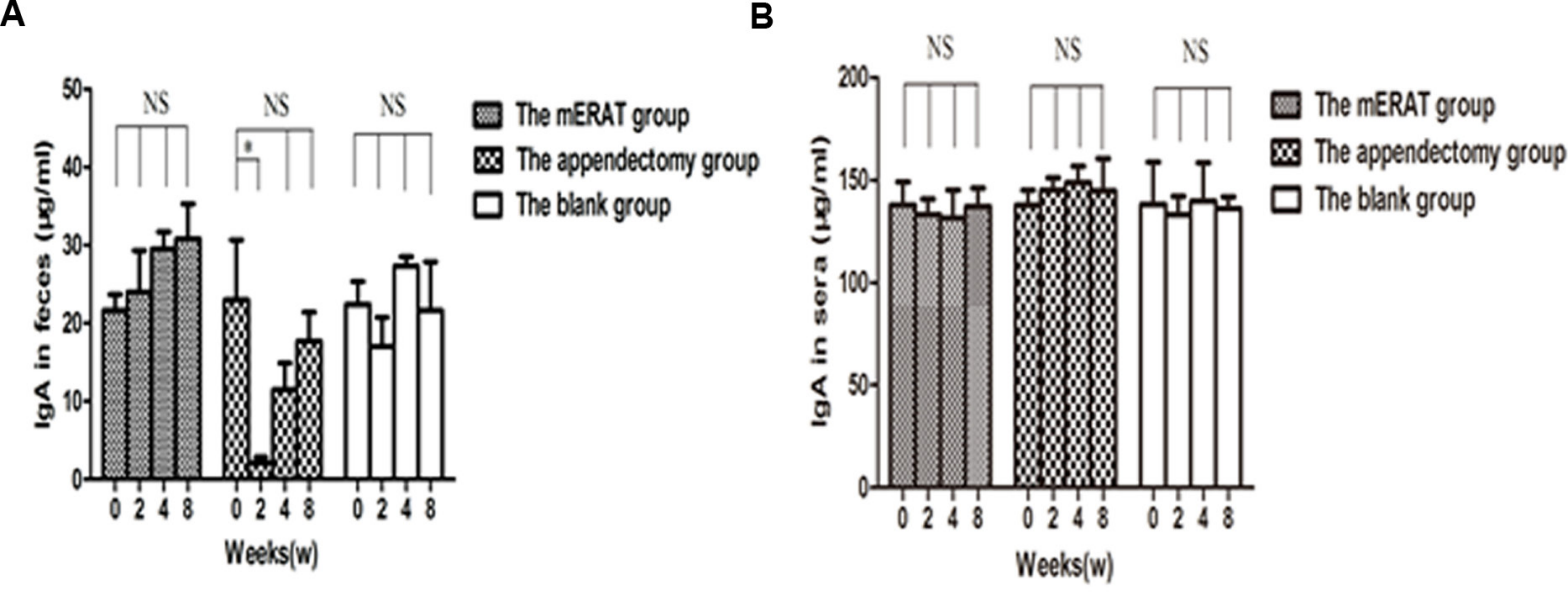
Levels of IgA in feces and sera before modeling and at 2, 4, 8 weeks after treatment **A.** levels of IgA in feces. **B.** levels of IgA in sera.

**Figure 4:**
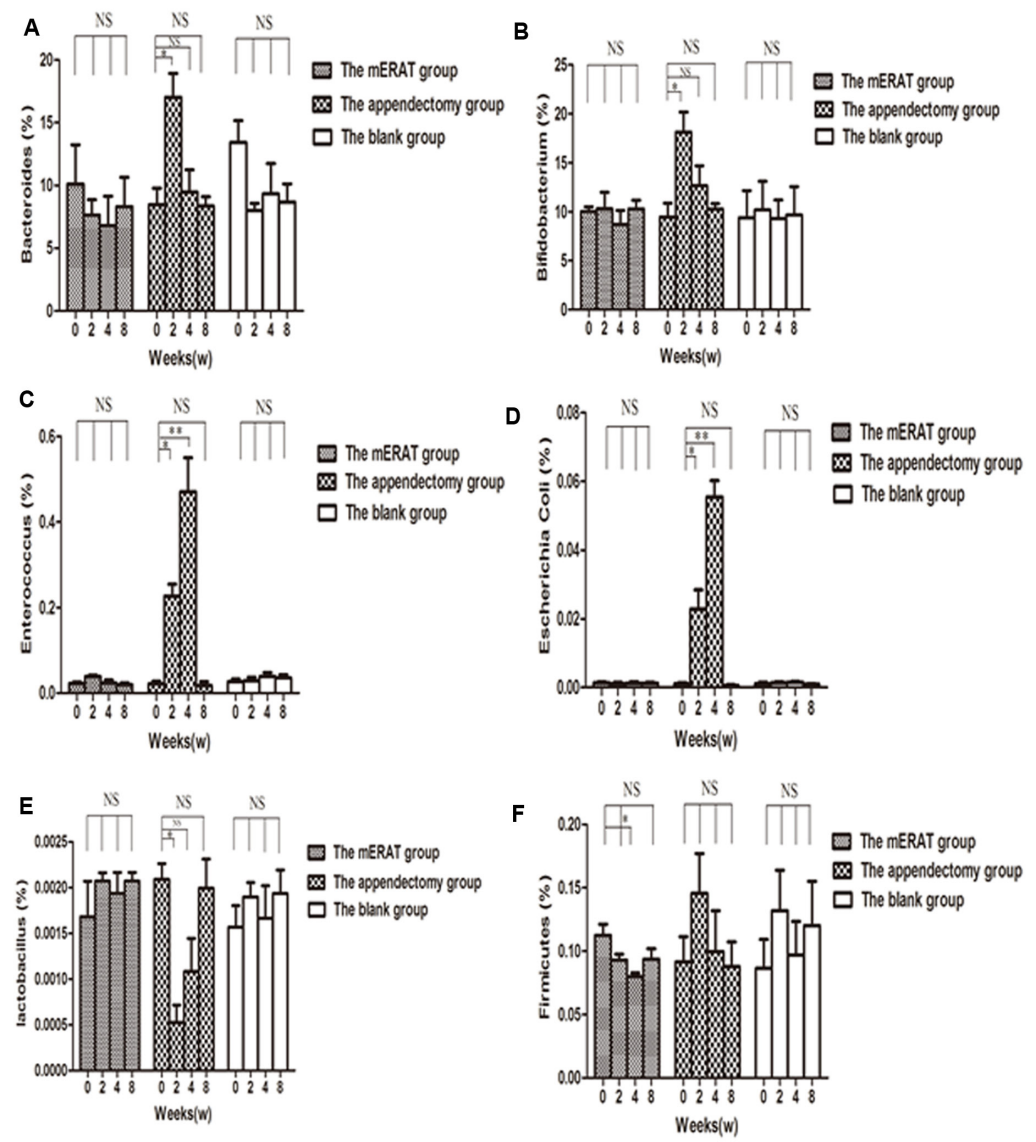
The composition of fecal microbiota before modeling and at 2, 4, 8 weeks after treatment in rabbits of the mERAT group, the appendectomy group and the blank group **A.**-**F.** Quantitative real-time PCR was used to determine fecal microbiota in rabbits of the three groups. Values are shown as a relative ratio to total bacterial 16s rRNA measured by 2^-∆∆ct^ method. **A.**-**F.** *P < 0.05, **P < 0.01, NS = no significance.

Levels of IgA in feces and sera did not change before modeling (0 week), and at 2, 4, 8 weeks after mERAT in group I (*P >* 0.05). However, levels of IgA in feces were lower than the original level at 2 weeks after appendectomy, but not in sera (Figure 3A, 3B). The middle section of proximal colon, distal colon and cecum were sampled to represent large intestine (Supplementary Figure 1A-1C). The number of IgA^+^ cells in the large intestine did not change before modeling, and at 2, 4, 8 weeks after treatment in group I and II (Supplementary Figure 2). The composition of fecal microbiota has scarcely changed before and after mERAT in group I, but firmicutes decreased at 4 weeks after mERAT (Figure 4F). However, fecal microbiota changed greatly at 2 and 4 weeks after appendectomy. Bacteroides and bifidobacterium increased at 2 weeks after appendectomy (Figure 4A, 4B); enterococcus and escherichia coli went up 2 and 4 weeks later (Figure 4C, 4D). Lactobacillus went down at 2 weeks after appendectomy, while firmicutes almost kept unchanged (Figure 4E, 4F). Subsequently, all fecal microbiota recovered to their original level in 8 weeks.

**Figure 5:**
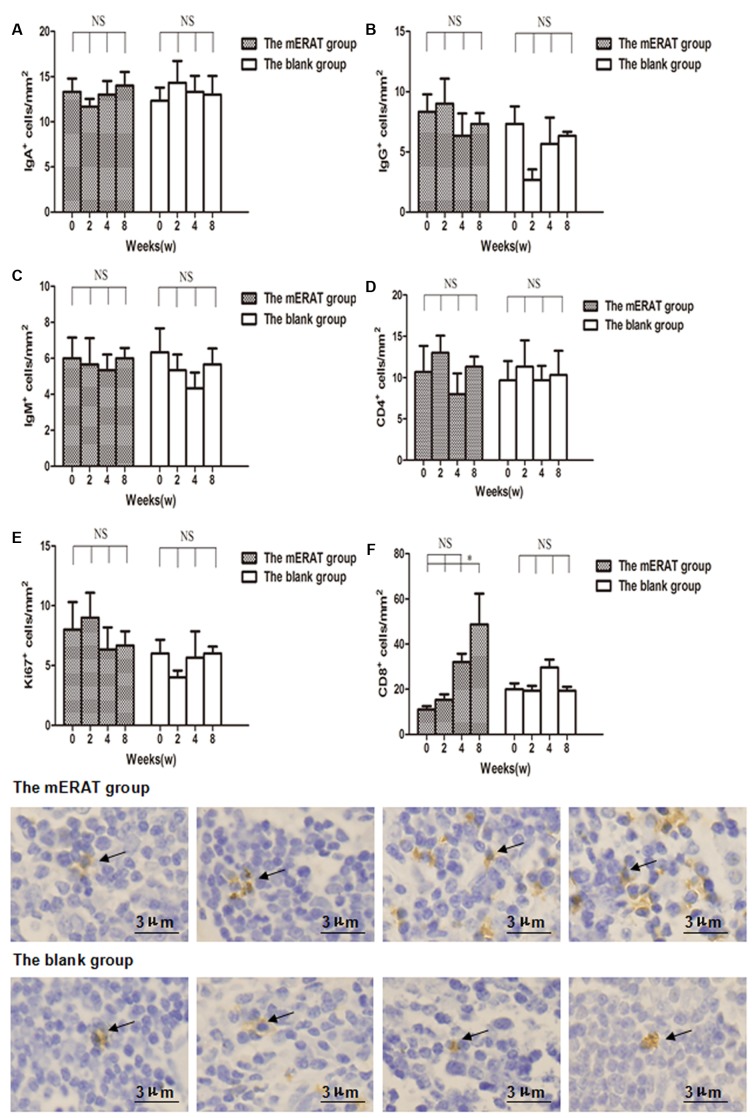
The number of IgA^+^, IgG^+^, IgM^+^, CD4^+^, Ki67^+^, and CD8^+^ cells in the appendix before modeling and at 2, 4, 8 weeks after treatment in rabbits of the mERAT group and the blank group **A.** The number of IgA^+^ cells. **B.** The number of IgG^+^ cells. **C.** The number of IgM^+^ cells. **D.** The number of CD4^+^ cells. **E.** The number of Ki67^+^ cells. **F.** The number of CD8^+^ cells; CD8^+^ cells in the appendix before modeling and at 2 ,4 , 8 weeks after mERAT (from left to right, black arrow); CD8^+^ cells in the appendix in the blank group at the same time point as the mERAT group (from left to right, black arrow). (A-F) *P < 0.05, NS = no significance. Bar = 3μm.

**Figure 6:**
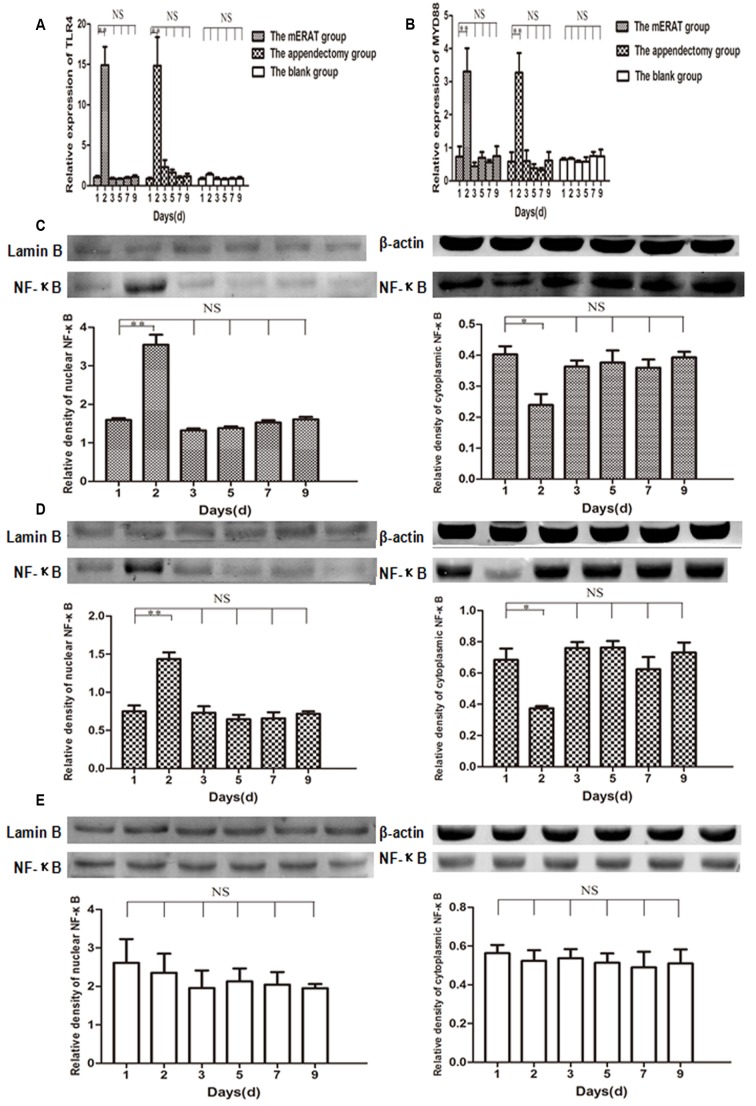
Relative expression of TLR4, MYD88, and NF-κB on the 1st, 2nd, 3rd, 5th, 7th, 9th day in rabbits of the mERAT group, the appendectomy group and the blank group **A.** Relative mRNA expression of TLR4 was detected by qPCR. **B.** Relative mRNA expression of MYD88. **C.** Relative expression of NF-κB in nuclear and cytoplasmic protein was determined by western blot in rabbits of the mERAT group. LaminB (64KDa), β-actin (42KDa), NF-κB p65 (60KDa). **D.** Relative expression of NF-κB in nuclear and cytoplasmic protein in rabbits of the appendectomy group. **E.** Relative expression of NF-κB in nuclear and cytoplasmic protein in rabbits of the blank group.

There were no obvious differences in the number of IgA^+^, IgG^+^, IgM^+^, CD4^+^, and Ki67^+^ cells in appendix before modeling, and at 2, 4, 8 weeks after treatment between group I and IV, but CD8^+^ cells of group I increased gradually within 8 weeks (Figure 5A-5F, Supplementary Figure 3A-3E). Relative expression of TLR4, MYD88, and NF-κB increased on the 2^nd^ day, and returned to the original level on the 3^rd^ day in rabbits of group I and II (Figure 6A-6E). But they did not change with immune parameters before modeling, and at 2, 4, 8 weeks after treatment in group I and II (Supplementary Figure 4A-4E).

## DISCUSSION

As a peripheral lymphoid organ, the appendix has abundant lymphoid tissue. It was reported that IgA levels in sera and colon decreased significantly when people of different ages received appendectomy [26]. Randal Bollinger R proposed that the human appendix is well suited as a ‘safe house’ for commensal bacteria, providing support for bacterial growth [22]. In 2014, an animal experiment demonstrated that IgA-secreting cells migrated to the large and small intestines from appendix, playing a significant role in the balance of intestinal bacteria [21]. A recent study has also ensured the role of appendix in gut immune responses as well as gut homeostasis [23]. However, nobody has studied the impact of mERAT and appendectomy on rabbits’ immune function after acute suppurative appendicitis and determined whether TLR4/MYD88/NF-κB signaling pathway was activated in this process.

WBC count and preoperative elevated CRP levels can aid the diagnosis of acute appendicitis. According to literatures, the overall sensitivity of CRP ranges from 40% to 99% with a specificity of 27-90% [27]. TNF-α and IL-6 are pro-inflammatory cytokines. IL-4 and IL-10 are anti-inflammatory cytokines. In our study, the WBCs decreased, while CRP, TNF-α, IL-6, IL-4, and IL-10 increased after successful modeling and returned to the original level after treatment. The results indicated that both mERAT and appendectomy can alleviate inflammation reaction by inhibiting cytokine production and that there was no significant difference in the treatment of acute suppurative appendicitis between the two methods. Therefore, the results may indicate that ERAT is an effective way for acute appendicitis.

IgA contains serum IgA and secretory IgA (SIgA). Intestinal SIgA can protect intestine from pathogens and toxins, contributing to the activation of mucosal immunity and the maintenance of gut homeostasis [28]. IgA-secreting cells develop in gut-associated lymphoid tissues (GALTs). Interestingly, we found that levels of sera IgA did not change before modeling, and at 2, 4, 8 weeks after mERAT or appendectomy. Levels of SIgA in feces remained about the same, while firmicutes decreased at 4 weeks after mERAT. However, the concentrations of SIgA in feces were lower at 2 weeks after appendectomy. Fecal microbiota changed dramatically at 2 and 4 weeks after appendectomy. SIgA in feces and fecal microbiota returned to the original level at 8 weeks after appendectomy. The number of IgA^+^ cells in the large intestine showed no change before modeling, and at 2, 4, 8 weeks after treatment in group I and II. Nevertheless, the result is different from a previous study. It was reported that the number of IgA^+^ cells in the large intestine was markedly decreased in appendectomized mice at 2 weeks as well as 4 weeks [21]. The reasons for the difference may be that different animal species and experimental conditions were used in the two studies. Our results may indicate that the two methods did not have impact on systematic immune function. The phenomenon may also suggest that mERAT had a slight impact on intestinal immune function of rabbits, while appendectomy damaged intestinal immune function to some extent. We speculated that the lack of appendix is bad for intestinal immune function, but other GALTs may compensate its function in 8 weeks. In our study, although the number of IgA^+^ cells in the large intestine did not change, the function of the cells may be influenced by appendectomy. According to the results, we deduced that ERAT may not cause damage to intestinal immune function, but appendectomy does have negative effect on it. Thus, ERAT is regarded as a better way for acute uncomplicated appendicitis.

B cells and T cells are involved in immune response. Ki67 is closely related to mitosis and indispensable in cell proliferation. B cell subsets (IgA^+^, IgG^+^, IgM^+^), T cell subsets (CD4^+^, CD8^+^), and Ki67^+^ cells in appendix were determined by immunohistochemistry. There were no obvious differences in the number of IgA^+^, IgG^+^, IgM^+^, CD4^+^, and Ki67^+^ cells in appendix before modeling, and at 2, 4, 8 weeks after treatment between the mERAT group and the blank group, but CD8^+^ cells of the mERAT group increased gradually within 8 weeks. Since antibiotics were not used in this study, the reasons for the increase of CD8^+^ cells may be that they were needed for killing the infected cells in the appendix. Our results implied that mERAT had a slight impact on immune function of appendix, and that proliferation of appendix was hardly affected. Hence, we conjectured that the immune function and proliferation of appendix of patients were seldomly affected by ERAT.

Toll-like receptors (TLRs) are the most important pattern recognition receptors in innate immunity [29]. TLR4 recognizes lipopolysaccharides from gram-negative bacteria [30]. It was reported that increased epithelial TLR4 expression induced intestinal microbiota changes in mice [31]. In the present study, relative expression of TLR4, MYD88, and nuclear NF-κB increased in acute phase of inflammation, but they did not change with immune parameters in the mERAT group and the appendectomy group. It may suggest that TLR4/MYD88/NF-κB signaling pathway was activated in acute inflammation, but that it did not participate in the process of regulating rabbits’ intestinal immune function.

There are some limitations in this study. First, the rabbits were executed within 8 weeks, so recurrences and long-term complications of mERAT can not be observed here. Second, it is to be studied whether stimulus to rabbits in the appendectomy group causes immune parameters changes. Third, further studies are needed to shed light on the mechanism of intestinal immune function changes after appendectomy. Therefore, we will focus on these issues in our further studies.

In conclusion, both mERAT and appendectomy are effective ways for acute suppurative appendicitis. mERAT was more preferable due to its advantage in maintaining intestinal immune funtion of rabbits. TLR4/MYD88/NF-κB signaling pathway was activated in acute phase of appendicitis.

## MATERIALS AND METHODS

### Animals

48 Japanese big-ear rabbits, weighing 2.5-3 kg and 5-6 months old, regardless of gender, were used in this study. The rabbits were raised in the animal research facility at the 2nd Affiliated Hospital of Harbin Medical University. The research was performed with the approval of the Animal Care and Use Committee of the 2nd Affiliated Hospital of Harbin Medical University. All animal experiments were performed in accordance with the NIH Guide for the Care and Use of Laboratory Animals.

### Groups

48 rabbits were randomly assigned into 4 groups : group I, the mERAT group; group II, the appendectomy group; group III, the model group; and group IV, the blank group. Appendix ligation was performed on rabbits of the first three groups to make acute suppurative appendicitis models. Then, mERAT was performed on rabbits of group I, while rabbits of group II underwent appendectomy. Laparotomy was conducted on rabbits of group III without treatment. Rabbits of group IV did not receive any surgery.

### Anesthesia

The rabbits fasted for 8 hours and water was prohibited 4 hours prior to the procedure. General anesthesia was accomplished with intravenous 3% pentobarbital sodium ( 1 ml/kg )( merck, Germany ).

### Animal models

The rabbits were placed in a supine position on the operation table. A 4 cm long ventral midline incision was made after skin preparation and sterilization. The appendiceal artery and vein were carefully separated to ensure blood supply of the appendix. Then, a sterile rubber band was used to ligate the appendix root through mesoappendix (Supplementary Figure 5A, 5B) [32]. Then, appendix was returned back and the incision was sutured. The rabbits showed symptoms of body temperature changes (anal temperature > 40 ^°^C or < 38^°^C), listlessness, anorexia, and abdominal distention progressively. The appendix was swollen with high tension because mucus and pus were accumulated in the lumen 24 hours after ligation (Supplementary Figure 5C). The pathology of appendix was confirmed as acute suppurative appendicitis, which meant models were successfully made (Supplementary Figure 5D).

### mERAT

The abdomen was opend along the original incision. The appendix was found and a small incision was made at the end of the appendix to drain mucus and pus. The appendiceal lumen was washed off with normal saline repeatedly by using a catheter (PR-104Q-1, Olympus, Japan). The guidewire (Boston Scientific, US) was pushed across the ligation through the small incision and a self-expanding metallic stent (10mm×40mm, Micro-tech Nanjing Co., Ltd, China) was placed in the lumen over the guidewire to relieve obstruction (Supplementary Figure 5E). The incision of the appendix and abdominal cavity was closed finally.

### Appendectomy

The appendix was found. The appendiceal vessels were ligated and cut off. The root of appendix was wrapped with a saline gauze to prevent intraoperative contamination. The appendix was lifted and a purse string suture was performed on the cecal wall around the root of appendix. The root of appendix was ligated. Then, the appendix was cut off (Supplementary Figure 5F). The appendiceal stump was sterilized and embedded. The incision of the abdomen was closed.

### Postoperative follow-up

All rabbits were given water 6 hours after treatment and a regular diet 24 hours after treatment. The animals were carefully monitored everyday. Blood samples were collected before modeling (marked as the 1^st^ day, and 0 week), after successful modeling (the 2^nd^ day), on the 3^rd^, 5^th^, 7^th^, 9^th^ day, and at 2, 4, 8 weeks after treatment. 3 rabbits in the first, second and forth groups were executed randomly before modeling (0 week) and at 2, 4, 8 weeks after treatment to get feces, colon, and appendix.

### Hematimetry

The WBC count was detected using XE-2100D blood-counter system (SYSMEX Corporation, Japan).

### Elisa

Levels of CRP, TNF-α, IL-6, IL-4, and IL-10 were determined using rabbit ELISA kits (Cloud-Clone Corp, USA). Fecal samples were homogenized in 1× diluent concentrate (10 mg feces in 1ml 1× diluent concentrate) and centrifuged stepwise with increasing force (400 g for 5min, 8,000 g for 10 min and 19,000 g for 10 min) to get rid of debris [21]. Levels of IgA in feces and sera were tested by a rabbit IgA ELISA kit (Abcam, UK). Optical densities were determined at a wavelength of 450nm.

### Quantitative real-time PCR (qPCR)

Total RNA was extracted using RNAsimple Total RNA Kit (TIANGEN BIOTECH CO., LTD, China)*.* Total RNA was reverse transcribed into complementary DNA (cDNA) with All-in-one^TM^ First-Strand cDNA Synthesis Kit (GeneCopoenia, USA). The cDNA was analysed by qPCR with SYBR GREEN qPCR Master Mix (Sangon Biotech, China) and an ABI 7500 fast system (Applied Biosystems, USA). Amplification conditions were: 50^°^C (2min), 95^°^C (10 min), 40 cycles of 95 ^°^C (15 s) and 60 ^°^C (60 s), 95 ^°^C (15 s). The cDNA was amplified in 20 μl reaction system. Feces were collected in sterile tubes when rabbits were dissected. Bacterial DNAs from feces were extrated using a feces DNA extraction kit (TIANGEN BIOTECH CO., LTD, China). Fecal microbiota was identified by qPCR. The primers can be found in Table 1.

**Table 1:** Group and species-specific primers used in this study.

Target organism	Sequence 5’-3’	Amplication size	References
β-actin	F:TGGCTCTAACAGTCCGCCTAG	275bp	This study
	R:AGTGCGACGTGGACATCCG		
TLR4	F:AAGGCAACTCGGATGTGAG	137bp	This study
	R:TGTGGGCTTAGAACAACTGG		
MYD88	F:GTGATGAACCGCAGGATACTG	131bp	This study
	R:CAGAGCAAGGAGTGTGACTTC		
All bacteria	F:CGGTGAATACGTTCCCGG	147bp	Suzuki et al [33].
	R:TACGGCTACCTTGTTACGACTT		
Bacteroides	F:GAGAGGAAGGTCCCCCAC	100bp	Wei F [34].
	R:CGCTACTTGGCTGGTTCAG		
Bifidobacterium	F:TCGCCTCCGGGTGAGAGTGG	198bp	This study
	R:CGAAGCCATGGTGGGCCGTT		
Firmicutes	F:GCTGCTAATACCGCATGATATGTC	81bp	Wei F [34].
	R:CAGACGCGAGTCCATCTCAGA		
Enterococcus	F:CCCCAAGAGTCCACATCG	282bp	This study
	R:GCGTTTATCCCTTCCCTAC		
Lactobacillus	F:AGCAGTAGGGAATCTTCCA	375bp	This study
	R:CGCCACTGGTGTTCYTCCATATA		
Escherichia Coli	F:CATGCCGCGTGTATGAAGAA	96bp	Huijsdens et al [35].
	R:CGGGTAACGTCAATGAGCAAA		

### Western blot analysis (WB)

Lymphocytes were prepared from peripheral blood using a lymphocyte separation medium kit (Tianjinhaoyang Biological Manufacture CO.,LTD, China). Cytoplasmic and nuclear protein from lymphocytes were extracted using a protein extraction kit (Beyotime Biotechnology, China). Protein concentrations were determined by BCA protein assay kit (Beyotime Biotechnology, China). Proteins were fractionated by SDS-PAGE and electrotransferred onto polyvinylidene difluoride membranes. The membrane was then blocked with 5% non-fat powdered milk (Sangon Biotech, China) for 1.5 h, followed by incubation with a primary antibody at 4°C overnight. The primary antibodies were listed in Table 2. After washed by phosphate buffer saline (PBS) with Tween-20, the membrane was incubated with IRDye 800CW Goat anti-Rabbit IgG (H + L) secondary antibody (1/5000, LI-COR Biosecience, UK) for 1 h. Then the antigen-antibody complexes were determined by infrared imaging system (Odyssey; LI-COR Biosecience, UK).

**Table 2:** Primary antibodies used for western blot and immunohistochemistry.

Antibody	Company (Cat. No.)	Working dilutions
β-actin	Biosynthesis biotechnology (bs-0061R)	WB: 1/1500
Lamin B	Biosynthesis biotechnology (bs-20349R)	WB: 1/500
NF-κB p65	Abcam (ab90532)	WB: 1/1000
IgA	Abcam (ab97186)	IHC: 1/400
IgG	Abcam (ab190492)	IHC: 1/50
IgM	Abcam (ab97191)	IHC: 1/400
CD4	Novus (NBP1-19371)	IHC: 1/50
CD8	Novus (NB100-64021)	IHC: 1/10
Ki67	Abcam (ab15580)	IHC: 1/400

### Immunohistochemical staining (IHC)

The tissues were fixed with 4% paraformaldehyde solution, which were then embedded in paraffin and serially cut for histological examination. Then, tissue sections were treated with 3 % H_2_O_2_ for 10 min after dewaxing and hydration. Subsequently, antigen retrieval was performed under high pressure using PH 6.0 sodium citrate buffer for 3 min. After that, tissue sections were incubated with primary antibody at 4°C overnight. The primary antibodies were listed in Table 2. Then tissue sections were treated with secondary antibody for 20 min at room temperature, determined by DAB detection kit (Zhongshan Jinqiao Biotechnology, China). Nuclei were counterstained with hematoxylin. The tissue sections were analyzed using a microscope (Olympus, Japan).

### Statistical anylysis

Measurement data were presented as mean ± standard deviation (SD). The data that contains two or three groups in each variable were analyzed by repeated measurements using SAS 9.3. In all analyses, *P* < 0.05 was considered significant.

## SUPPLEMENTARY MATERIALS FIGURES



## Abbreviations

ERAT: endoscopic retrograde appendicitis therapy
mERAT: mimic endoscopic retrograde appendicitis therapy
CRP: C-reactive protein
TNF-α: tumor necrosis factor α
IL-6: interleukin-6
IL-4: interleukin-4
IL-10: interleukin-10
qPCR: quantitative real-time PCR
WB: western blot
IHC: immunohistochemical Staining
SD: standard deviation
WBC: white blood cell
SIgA: secretory IgA
GALTs: gut-associated lymphoid tissues
TLRs: Toll-like receptors
