# Supplementary material for: The immune impact of mimic endoscopic retrograde appendicitis therapy and appendectomy on rabbits of acute appendicitis
Source: Oncotarget. 2017 Mar 15;8(39):66528–39. doi: 10.18632/oncotarget.16236 (PMC5630433; doi:10.18632/oncotarget.16236)
Supplement: Supplementary file 1 [file oncotarget-08-66528-s001.pdf]

The immune impact of mimic endoscopic retrograde appendicitis therapy and appendectomy on rabbits of acute appendicitis

Supplementary Material

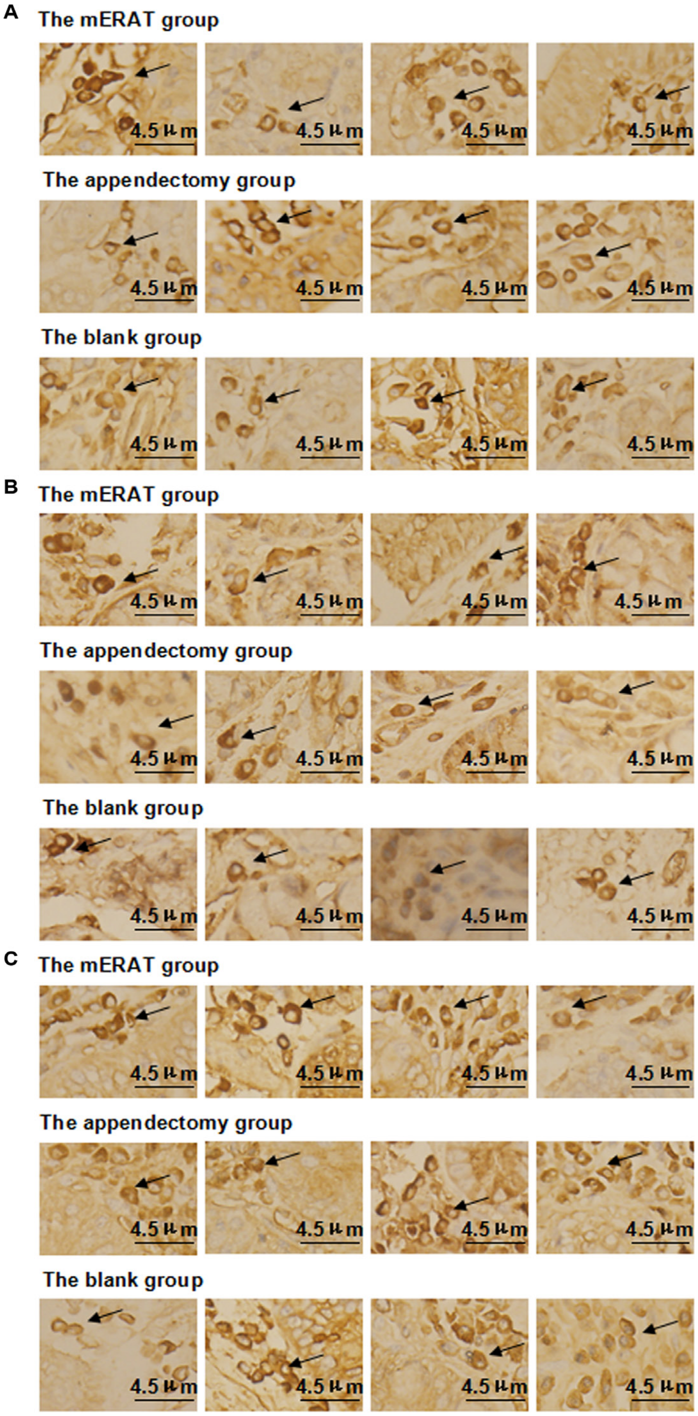

Supplementary Figure 1: IgA+ cells in the proximal colon, distal colon and cecum before modeling and at 2, 4, 8 weeks after treatment in rabbits of the mERAT group, the appendectomy group and the blank group. (A) IgA+ cells in the proximal colon (from left to right, black arrow). (B) IgA+ cells in the distal colon (from left to right, black arrow). (C) IgA+ cells in the cecum (from left to right, black arrow).

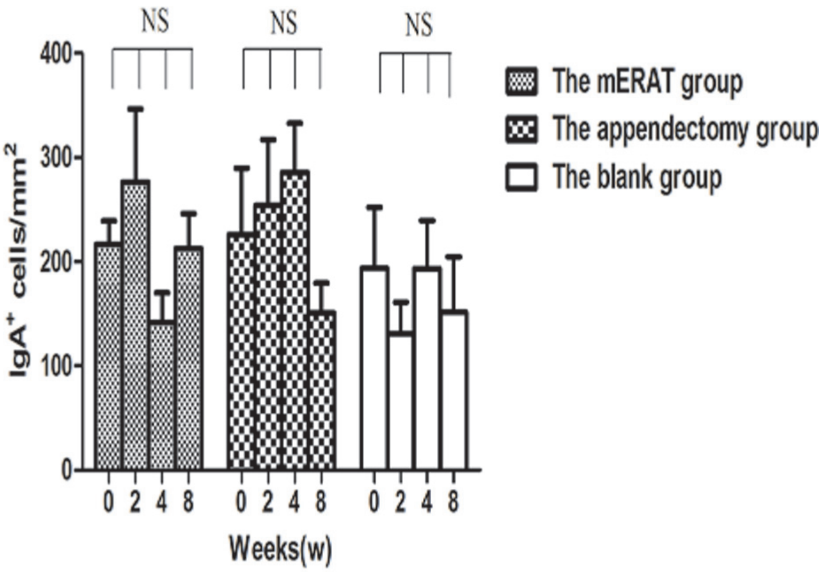

Supplementary Figure 2: The number of IgA+ cells in the large intestine before modeling and at 2, 4, 8 weeks after treatment. NS = no significance.

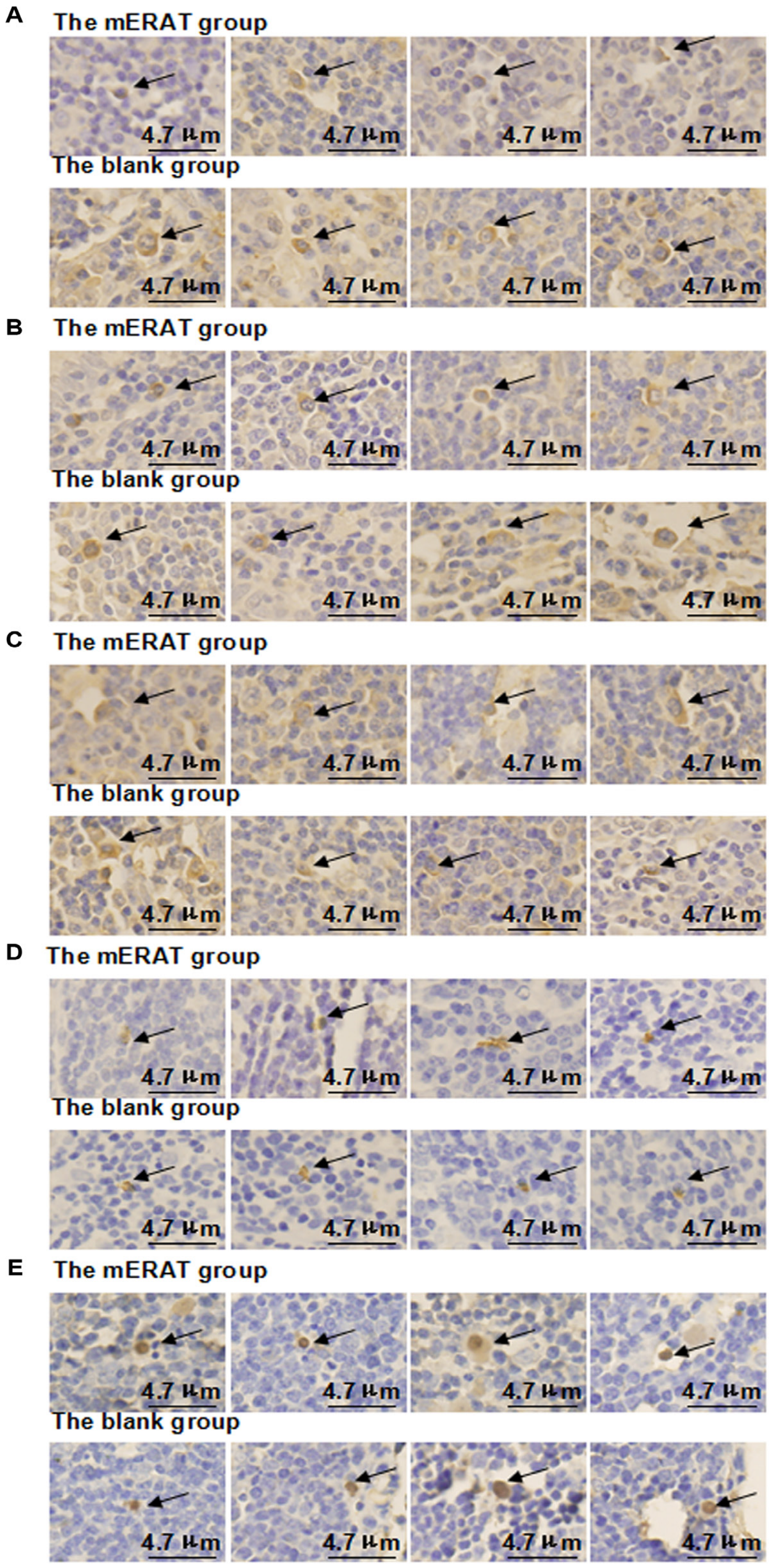

Supplementary Figure 3: IgA+, IgG+, IgM+, CD4+, and Ki67+ cells in the appendix before modeling and at 2, 4, 8 weeks after treatment in rabbits of the mERAT group and the blank group. (A) IgA+ cells in the appendix (from left to right, black arrow). (B) IgG+ cells in the appendix (from left to right, black arrow). (C) IgM+ cells in the appendix (from left to right, black arrow). (D) CD4+ cells in the appendix (from left to right, black arrow). (E) Ki67+ cells in the appendix (from left to right, black arrow).

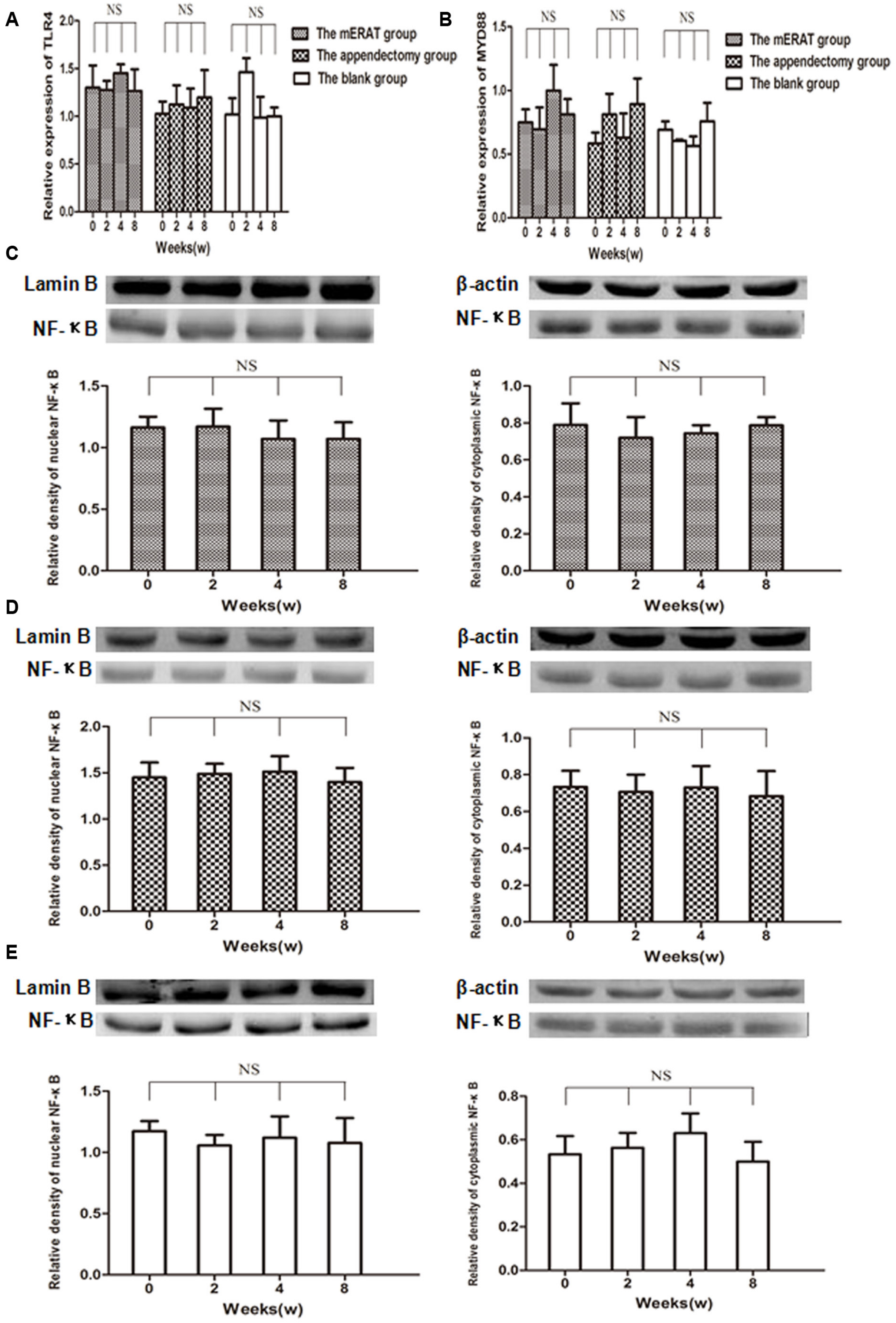

Supplementary Figure 4: Relative expression of TLR4, MYD88, and NF-κB before modeling and at 2, 4, 8 weeks after treatment in rabbits of the mERAT group, the appendectomy group and the blank group. (A) Relative mRNA expression of TLR4. (B) Relative mRNA expression of MYD88. (C) Relative expression of NF-κB in nuclear and cytoplasmic protein in rabbits of the mERAT group. (D) Relative expression of NF-κB in nuclear and cytoplasmic protein in rabbits of the appendectomy group. (E) Relative expression of NF-κB in nuclear and cytoplasmic protein in rabbits of the blank group.

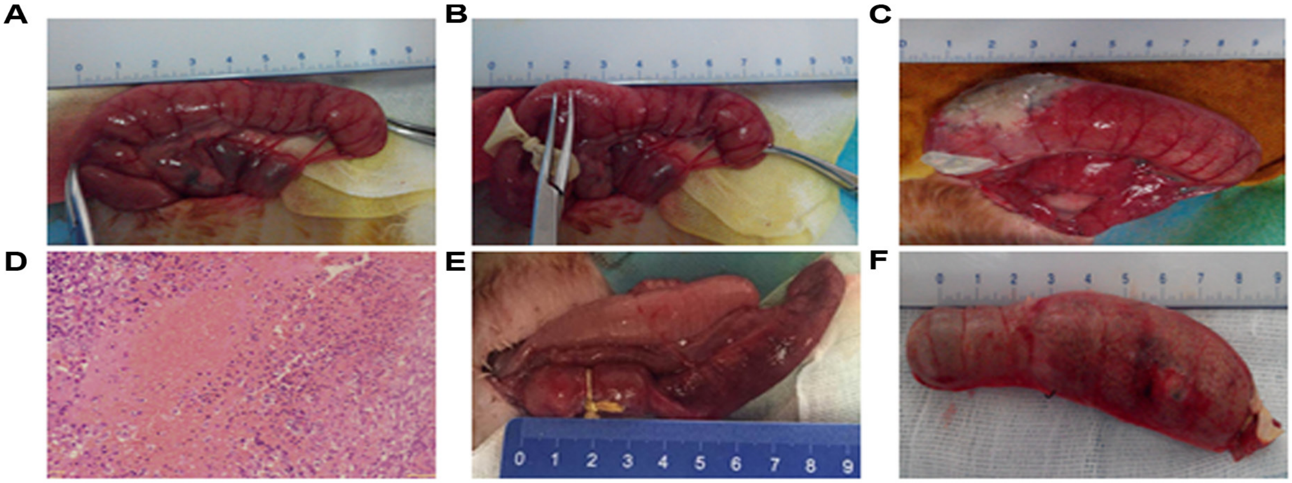

Supplementary Figure 5: Photos of the procedures in rabbits of the mERAT group, the appendectomy group, and histological examination. (A) The appendix of a normal rabbit. (B) The appendix was ligated at its root with a sterile rubber band. (C) The appearance of acute suppurative appendicitis. (D) The appendix tissue was examined by haematoxylin-eosin staining (Bar = 32μm). (E) A self-expanding metallic stent was placed in the appendix. (F) The appendix after appendectomy.
